# Risk-adjusted operative delivery rates and maternal-neonatal outcomes as measures of quality assessment in obstetric care: a multicenter prospective study

**DOI:** 10.1186/s12884-015-0450-2

**Published:** 2015-02-05

**Authors:** Gianpaolo Maso, Lorenzo Monasta, Monica Piccoli, Luca Ronfani, Marcella Montico, Francesco De Seta, Sara Parolin, Caterina Businelli, Laura Travan, Salvatore Alberico, on behalf of The Multicenter Study Group on Mode of Delivery in Friuli Venezia Giulia

**Affiliations:** Department of Obstetrics and Gynecology, Institute for Maternal and Child Health – IRCCS Burlo Garofolo, Via dell’Istria 65/1, Trieste, 34137 Italy; Epidemiology and Biostatistics Unit, Institute for Maternal and Child Health – IRCCS Burlo Garofolo, Trieste, Italy; Department of Obstetrics and Gynecology, Institute for Maternal and Child Health – IRCCS Burlo Garofolo and Department of Medical Sciences, University of Trieste, Trieste, Italy; Department of Neonatology and Neonatal Intensive Care, Institute for Maternal and Child Health – IRCCS Burlo Garofolo, Trieste, Italy

**Keywords:** Operative delivery, Risk adjustment, Quality of care, Maternal outcome, Neonatal outcome

## Abstract

**Background:**

Although the evaluation of caesarean delivery rates has been suggested as one of the most important indicators of quality in obstetrics, it has been criticized because of its controversial ability to capture maternal and neonatal outcomes. In an “ideal” process of labor and delivery auditing, both caesarean (CD) and assisted vaginal delivery (AVD) rates should be considered because both of them may be associated with an increased risk of complications.

The aim of our study was to evaluate maternal and neonatal outcomes according to the outlier status for case-mix adjusted CD and AVD rates in the same obstetric population.

**Methods:**

Standardized data on 15,189 deliveries from 11 centers were prospectively collected. Multiple logistic regression was used to estimate the risk-adjusted probability of a woman in each center having an AVD or a CD. Centers were classified as “above”, “below”, or “within” the expected rates by considering the observed-to-expected rates and the 95% confidence interval around the ratio. Adjusted maternal and neonatal outcomes were compared among the three groupings.

**Results:**

Centers classified as “above” or “below” the expected CD rates had, in both cases, higher adjusted incidence of composite maternal (2.97%, 4.69%, 3.90% for “within”, “above” and “below”, respectively; p = 0.000) and neonatal complications (3.85%, 9.66%, 6.29% for “within”, “above” and “below”, respectively; p = 0.000) than centers “within” CD expected rates. Centers with AVD rates above and below the expected showed poorer and better composite maternal (3.96%, 4.61%, 2.97% for “within”, “above” and “below”, respectively; p = 0.000) and neonatal (6.52%, 9.77%, 3.52% for “within”, “above” and “below”, respectively; p = 0.000) outcomes respectively than centers with “within” AVD rates.

**Conclusions:**

Both risk-adjusted CD and AVD delivery rates should be considered to assess the level of obstetric care. In this context, both higher and lower-than-expected rates of CD and “above” AVD rates are significantly associated with increased risk of complications, whereas the “below” status for AVD showed a “protective” effect on maternal and neonatal outcomes.

## Background

Quality of care is an important topic in modern obstetrics of which risk-adjusted caesarean delivery (CD) rate is often used as an indicator, with the implicit assumption that low rates may reflect evidence-based intervention [1-8].

Although the evaluation of risk-adjusted CD rates is an important factor in quality assessment, it is just one of the elements to be considered in the process of labor and delivery auditing. In this regard, a comprehensive assessment should encompass both maternal and neonatal outcomes according to mode of delivery [4]. Several studies focused on the association between institutional adjusted CD rates and outcomes reporting controversial results. In their retrospective cohort study on 748,604 low risk singleton pregnancies, Gould *et al.* observed that neonatal morbidity (birth asphyxia and intensive care-therapeutic interventions) was increased both in low- and high-CD rate hospitals [5]. Bailit *et al.*, considering the Washington State Birth Events Records for 1995 and 1996, showed that asphyxiated infants were likely to be delivered by caesarean in hospitals in which CD rates were above the predicted range [6]. In another study, the same authors showed a mixed picture for hospitals with CD rates above the expected, with some poorer and some improved maternal and neonatal outcomes [7]. Srinivas *et al.* evaluated both maternal and neonatal composite outcomes according to institutional adjusted CD rate in a population-based cohort from 401 hospitals. Their conclusion was that lower-than-expected risk-adjusted CD rates were associated with an increased risk of maternal or neonatal complications and that above than expected risk-adjusted CD rates did not result in improved outcomes [8].

All the above mentioned studies have however limited their attention to the CD rate. None of them has evaluated the association between the risk of adverse maternal and neonatal outcomes and the outlier status for both adjusted caesarean and assisted vaginal delivery rates (AVD) in the same obstetric population. Including the rate of assisted vaginal delivery in this analysis may be crucial in the assessment of quality of care. In fact, institutions with low frequencies of risk-adjusted CD rates might have, as a balance, high adjusted AVD rates, potentially associated with adverse outcomes [9]. Limiting the evaluation of the obstetrics performance to the CD rates could therefore be misleading and not reflect the true outcomes of that center.

The aim of our study, carried out on more than 15,000 deliveries of 11 different centers of Friuli Venezia Giulia, a north-eastern region of Italy, was to determine the prevalence of adverse maternal and neonatal outcomes according to the mode of delivery. We tested the hypothesis that institutions with risk-adjusted AVD and CD rates above or below the expected would have higher and lower rates, respectively, of maternal and neonatal complications.

## Methods

We prospectively collected data on all deliveries occurring in the 11 hospitals of Friuli Venezia Giulia in a period of 18 months between July 2006 and December 2007. Friuli Venezia Giulia is a region of North-Eastern Italy accounting roughly for 10,000 deliveries per year with one of the lowest overall regional CD rate in Italy (23.4% in 2010). Virtually all births of the region were included in the study, given the very low rate of home births and the absence of midwifery-led centers in the area. The Institutions of the region, referred to as A to M, are level one units, serving low risk pregnancies, with the exception of centers I and M that are level three units (range 369–1,810 deliveries/year/unit).

To eliminate the potential bias generated by different definitions and heterogeneous collection of data, we created a regional computerized database considering maternal characteristics (maternal age and pre-pregnancy body mass index-BMI), variables related to pregnancy (parity, gestational age at delivery, singleton or multiples, presence of previous CD), antenatal clinical risk factors, mode of delivery and short term neonatal and maternal outcomes. Data on pregnancies were prospectively collected at the time of delivery and before maternal/neonatal discharge and were systematically reviewed every month by the referent obstetrician of each center.

Special attention was devoted to completeness and accuracy of data. During the study period, two of the authors (GM and SA) organized periodical multicenter meetings to discuss the results and provide assistance. The study was approved by the institutional review board of the coordinating center (Institute for Maternal and Child Health – IRCCS Burlo Garofolo, Trieste, project 86/05 – February 28, 2007) and access to the data was approved by all hospital trust administrations. According to the Italian law on privacy, data were anonymized at every institution where each patient was assigned a unique identifier.

Short term maternal and neonatal complications were analyzed both as single and combined complications (life threatening, non-life threatening and composite).

Life threatening maternal complications were defined as follow (criteria modified from McMahon [10]): 1. Major PPH (post-partum hemorrhage greater than 1000 mL or requiring blood transfusion) [11]; 2. Post-partum hysterectomy; 3. Obstetric wound hematoma requiring re-intervention; 4. Thromboembolic disease; 5. Uterine rupture. Non-life threatening maternal morbidities included: 1. Minor PPH (post-partum hemorrhage between 500 and 1000 mL) [11]; 2. III-IV degree perineal tears; 3. Asymptomatic wound dehiscence; 4. Endometritis or pyrexia needing antibiotic treatment; 5. Bowel or bladder injury; 6. Anaesthesiological complications; 7. Any other condition requiring admission to intensive care unit (ICU).

Life threatening neonatal complications (criteria modified from Fong [12]) included: 1. Mortality within 7 days of life; 2. Mortality within 28 days; 3. Abnormal neurologic status (encephalopathy as defined by Sarnat and Sarnat [13]), neonatal convulsions and intracranial hemorrhage (including all classes of intraventricular hemorrhage, epidural hemorrhage, and subdural hemorrhage). Non-life threatening neonatal morbidities were assessed as follow: 1. Pulmonary disorders, including transient tachypnoea of the newborn and respiratory distress syndrome, as defined by Hjalmarson [14]; 2. Bacterial infections including pneumonia and sepsis, diagnosed clinically with or without confirmation by blood cultures; 3. Umbilical artery cord pH at birth less than 7.00; 4. Umbilical artery cord base deficit greater than 12 mmol/L at birth; 5. Apgar score less than 7 at five minutes in term newborns; 6. Any other condition (birth trauma included) requiring neonatal intensive care (NICU) admission in term newborns for more than 24 hours (37–42 weeks/birth weight >2500 grams).

Incidence of complications was analyzed for all cases and divided into spontaneous vaginal (SVD), assisted vaginal (AVD), overall vaginal (VD) and caesarean deliveries (CD). Both women and newborns could have more than one complication, thus the total number of single complications is higher than the number of women or newborns with complications. In case of multiple pregnancies, if one of the newborns had a complication, this was considered as a neonatal complication. Only cases with complete data on all of the above indicated variables were included in the final analysis. Pregnancies complicated by antepartum stillbirths and/or life-threatening fetal congenital anomalies and deliveries with infants weighting less than 500 grams and/or below 24 weeks’ gestation were excluded to avoid potential bias in the evaluation of the outcomes.

Associations between type of delivery (CD vs. SVD, CD vs. VD and AVD vs. SVD) and single or composite complications were analyzed calculating crude and adjusted risk ratios (RRs) and p values, resulting from log-binomial regressions [15]. Considering that we had approximately 50 comparisons, we adopted a conservative Bonferroni correction dividing the significance level of 0.05 by 50: thus we considered p < 0.001 as statistically significant.

CD and AVD rates were adjusted for maternal age (reference 20–24 years, <20 years, 25–29 years, 30–35 years, >35 years), maternal pre-pregnancy BMI (reference 18.5-24.9 kg/m^2^, <18.5 kg/m^2^, 25 – 29.9 kg/m^2^, ≥30 kg/m^2^) [16], gestational age at delivery (reference 37–41 weeks, <30 weeks, 30–36 weeks, >41 weeks) classification of pregnancy at risk (reference no risk, low/intermediate risk, high risk), parity (reference multiparous, nulliparous), gestations (reference singleton, twin), presentation (reference cephalic, other), presence of previous CD (reference no past CD, one, more than one) newborn birth weight (reference 2,500-4,000 grams, <1,000 grams, 1,000-1,499 grams, 1,500-2,499 grams, >4,000 grams). Pregnancy was classified as at low-intermediate or high risk on the basis of the following definitions: 1. Low risk: if no pre-existing or ante partum risk factor was identified; 2. Intermediate risk: presence of pre-existing maternal medical conditions complicating the pregnancy, but not representing *per se* an absolute indication to CD or induction of labor (e.g. chronic hypertension, pregnancy-associated hypertension, gestational diabetes, obstetric cholestasis, polyhydramnios and Rh-isoimmunization); 3. High risk: presence of pre-existing maternal diseases or other obstetric conditions suggesting the need for delivery, such as HIV infection, pre-existing diabetes, severe pre-eclampsia, placenta previa, oligohydramnios and intrauterine growth restriction defined as fetal abdominal circumference or estimated fetal weight less than the 10^th^ centile [7]. In case of a multiple pregnancy, we considered the lowest newborn birth weight. Finally, given the acknowledged high risk of complications related to the delivery in the presence of impeding maternal and fetal compromise, the degree of urgency was also considered into the risk-adjustment (reference maternal and fetal compromise, no maternal and fetal compromise) [17].

Following these adjustments, we calculated for each of the 11 centers the expected AVD and CD rates.

According to the methodology adopted by Bailit *et al.*, a logistic regression model was initially developed to generate the predicted probability of operative deliveries (CD and AVD) for each patient. Second, the probabilities of operative deliveries for all patients were added together for each center to obtain the predicted number of CDs and AVDs for that institution. We then divided these predicted numbers of deliveries by the total number of patients who were delivered at that hospital to obtain the institutional expected caesarean and assisted vaginal delivery rates. Units were herein classified by evaluating the ratio of observed-to-expected rates and considering the 95% confidence interval (CI) around the ratio. If the 95% CI of the resulting ratio included 1, the center was classified as within the expected. If the 95% CI was above or below 1, the centers were respectively classified as above or below the expected [7]. Maternal and neonatal outcomes were thus analyzed according to the outlier status of the centers as within, above and below the expected rates. The incidences of maternal and neonatal complications were adjusted by maternal age, maternal BMI, pregnancy at risk (no, low, high), parity, fetal presentation, number of fetuses, presence of previous CD (no, one, more than one), gestational age at delivery and neonatal birth weight and delivery grade of urgency. Finally, given the potential influences of obstetric volume and the organization of newborn care on outcomes, complication rates were also adjusted by considering the number of deliveries per center (reference ≥1000 deliveries/year, <1000 deliveries/year) and the presence of a Neonatal Intensive Care Unit (reference available, non-available) [18-20]. Differences among adjusted outcomes were evaluated with the analysis of variance (ANOVA) with Bonferroni corrections for single comparisons between within vs. above and within vs. below the expected CD and AVD rates. Finally, considering we already had applied the correction to each outcome, we additionally corrected for the number of outcomes and considered as significant p values below 0.003.

All statistical analyses were performed using Stata/IC 11.2 software (StataCorp, College Station, TX, USA).

## Results

From a total number of 15,726 pregnancies, we excluded from the analysis cases with life-threatening fetal congenital anomalies (18 cases), all antepartum stillbirths (16) and incomplete records, regarding maternal age (18), BMI (441), classification of pregnancy at risk (10), neonatal complications (29) and maternal complications (5). Analyses were consequently carried out on 15,189 pregnancies.

Distributions of non-missing independent variables and CD/AVD rates were similar across the analyzed and the excluded records (data not shown).

**Figure 1:**
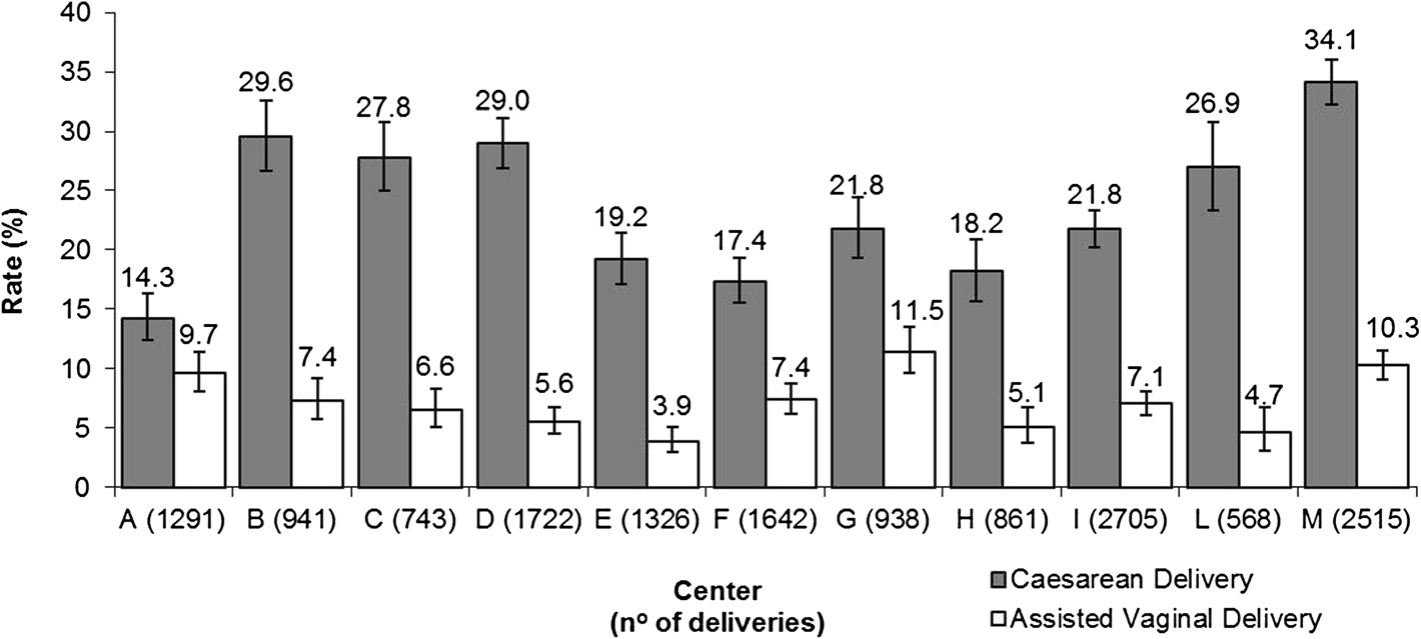
**Institutional caesarean and assisted vaginal delivery rates (percentage).** Centers are reported in capital letters.

CD and AVD rates by institution ranged from 14.3% to 34.1% and from 3.9% to 10.2% (Figure 1). Four hospitals (36.4%: B, D, L and M) had adjusted CD rates above the predicted confidence interval; four centers (36.4%: A, F, H, I) were below the interval and three centers (27.2%: C, E and G) fell within the interval for their patient population. With regard to AVD, two hospitals (18.3%: G and M) had adjusted rates above the predicted confidence interval; three (27.2%: E, H, L) were below the interval, and six (54.5%: A, B, C, D, F, I) were within the interval.

### Analysis of maternal and neonatal outcomes according to mode of delivery

The incidence and crude and adjusted RRs of maternal and neonatal outcomes according to mode of delivery are listed in Tables 1 and 2. Outcomes varied substantially by mode of delivery and some of them were obviously associated with only one mode of delivery (e.g. III-IV degree perineal tears). If a condition was inherent of a mode of delivery, then no comparative analysis was performed.

**Table 1:** **Incidence of outcomes (individual and composite) by mode of delivery**

	**Overall**	**SVD**	**CD**	**AVD**
	**n = 15,189**	**n = 10,410**	**n = 3,638**	**n = 1,141**
**Maternal complications**	***n (%)***	***n (%)***	***n (%)***	***n (%)***
Major PPH	61 (0.40)	30 (0.29)	20 (0.55)	11 (0.96)
Hysterectomy	8 (0.05)	3 (0.03)	5 (0.14)	0 (0.00)
Wound hematoma	49 (0.32)	30 (0.29)	9 (0.25)	10 (0.88)
TED	6 (0.04)	2 (0.02)	3 (0.08)	1 (0.09)
Uterine rupture	2 (0.01)	0 (0.00)	2 (0.05)	0 (0.00)
***Life threatening composite***	111 (0.73)	59 (0.57)	32 (0.73)	20 (1.75)
Minor PPH	369 (2.43)	232 (2.23)	90 (2.47)	47 (4.12)
III-IV degree tears	50 (0.33)	39 (0.37)	0 (0.00)	11 (0.96)
Wound Dehiscence	25 (0.16)	12 (0.12)	10 (0.27)	3 (0.26)
Endometritis	69 (0.45)	23 (0.22)	42 (1.15)	4 (0.35)
Bowel/bladder injury	4 (0.03)	0 (0.00)	4 (0.11)	0 (0.00)
Anaesthesiological	7 (0.05)	4 (0.04)	2 (0.05)	1 (0.09)
Other*	19 (0.13)	6 (0.06)	12 (0.33)	1 (0.09)
***Non-life threatening composite***	485 (3.19)	283 (2.72)	142 (3.90)	60 (5.26)
***Overall composite***	596 (3.92)	342 (3.29)	174 (4.78)	80 (7.01)
**Neonatal complications**	***n (%)***	***n (%)***	***n (%)***	***n (%)***
Mortality <7 days	17 (0.11)	3 (0.03)	13 (0.36)	1 (0.09)
Mortality <28 days	13 (0.09)	4 (0.04)	6 (0.16)	3 (0.26)
Neurologic symptoms	38 (0.25)	6 (0.06)	29 (0.80)	3 (0.26)
***Life threatening composite***	47 (0.31)	9 (0.09)	33 (0.91)	5 (0.44)
Pulmonary disorders	250 (1.65)	84 (0.81)	156 (4.29)	10 (0.88)
Bacterial infections	98 (0.65)	38 (0.37)	58 (1.59)	2 (0.18)
pH < 7.00	74 (0.49)	25 (0.24)	29 (0.80)	20 (1.75)
BD > 12 mmol/L	204 (1.34)	113 (1.09)	41 (1.13)	50 (4.38)
Apgar < 7	118 (0.78)	37 (0.36)	59 (1.62)	22 (1.93)
Other**	773 (5.09)	284 (2.73)	445 (12.23)	44 (3.86)
***Non-life threatening composite***	973 (6.41)	416 (4.00)	464 (12.75)	93 (8.15)
***Overall composite***	1,020 (6.72)	425 (4.08)	497 (13.66)	98 (8.59)

Footnotes: SVD, spontaneous vaginal delivery; AVD, assisted vaginal delivery; CD, caesarean delivery; VD vaginal delivery ; PPH, post-partum hemorrhage; TED, thromboembolic disease; BD, base deficit.

*Any other condition requiring Intensive Care Unit admission.

**Any other condition requiring Neonatal Intensive Care Unit admission in term neonates (37–42 weeks).

**Table 2:** **Risk ratios for outcomes (individual and composite) by mode of delivery**

	**CD vs. SVD**		**CD vs. VD**		**AVD vs. SVD**	
**Maternal complications**	***Crude RR***	***Adj RR***	***Crude RR***	***Adj RR***	***Crude RR***	***Adj RR***
Major PPH	1.91 (1.08-3.35)	0.81 (0.38-1.70)	1.55 (0.91-2.64)	0.52 (0.25-1.07)	3.35 (1.68-6.66)	2.41 (1.20-4.83)^§^
Hysterectomy	5.29 (1.27-22.13)	1.68 (0.27-10.54)	5.29 (1.27-22.13)	1.39 (0.44-8.16)	-	-
Wound hematoma	0.86 (0.41-1.81)	0.32 (0.07-1.48)	0.71 (0.35-1.47)	0.28 (0.07-1.14)	3.04 (1.49-6.20)	2.09 (0-97-4.51)
TED	4.29 (0.71-25.68)	0.31 (0.06-1.45)	3.18 (0.64-15.72)	0.45 (0.06-2.90)	4.56 (0.41-50.27)	2.10 (0.97-4.55)
Uterine rupture	-	-	-	-	-	-
***Life threatening composite***	1.57 (1.02-2.41)	0.61 (0.36-1.03)	1.30 (0.86-1.95)	0.49 (0.29-0.84)	3.18 (1.92-5.25)^§^	2.24 (1.46-.3.45)^§^
Minor PPH	1.11 (0.87-1.41)	0.66 (0.31-1.37)	0.71 (.129)	0.61 (0.28-1.32)	1.85 (1.36-2.51)^§^	1.41 (1.02-1.93)^§^
III-IV degree tears	-	-	-	-	3.47 (1.78-6.76)^§^	2.26 (1.13-4.52)^§^
Wound Dehiscence	2.38 (1.03-5.51)	1.15 (0.16-1.28)	2.12 (0.95-4.71)	0.97 (0.11-8.59)	2.28 (0.64-8.07)	2.14 (0.58-7.91)
Endometritis	5.22 (3.15-8.68)^§^	4.74 (2.53- 8.87)^§^	4.94 (3.05-8.00)^§^	4.33 (2.39-7.84)^§^	1.59 (0.55-4.58)	1.80 (0.79-4.08)
Bowel/bladder injury	-	-	-	-	-	-
Anaesthesiological	1.43 (0.26-7.81)	0.65 (0.16-2.55)	1.27 (0.25-6.54)	0.56 (0.12-2.46)	2.28 (0.26-20.39)	1.95 (0.21-18.03)
Other*	5.72 (2.15-15.24)^§^	1.59 (0.62-4.09)	5.44 (2.14-13.81)^§^	0.79 (0.40-1.55)	1.52 (0.18-12.62)	1.09 (0.12-9.76)
***Non-life threatening composite***	1.44 (1.18-1.76)^§^	0.85 (0.44-1.64)	1.32 (1.09-1.60)	0.92 (0.72-1.81)	1.96 (1.49-2.57)^§^	1.57 (1.11-2.23)^§^
***Overall composite***	1.46 (1.22-1.74)^§^	1.04 (0.61-1.80)	1.31 (1.01-1.56)	0.77 (0.39-1.53)	2.13 (1.69-2.70)^§^	1.67 (1.22-2.27)^§^
**Neonatal complications**	***Crude RR***	***Adj RR***	***Crude RR***	***Adj RR***	***Crude RR***	***Adj RR***
Mortality <7 days	12.40 (3.54-43.49)^§^	3.04 (0.65-14.31)	10.32 (3.37-31.63)^§^	2.50 (0.44-14.13)	3.04 (0.32-29.21)	3.61 (0.50-25.08)^§^
Mortality <28 days	4.29 (1.21-15.20)	0.68 (0.28-1.63)	2.72 (0.92-8.09)	0.51 (0.13-2.03)	6.84 (1.53-30.54)	7.12 (1.51-33.68)^§^
Neurologic symptoms	13.83 (5.75-33.28)^§^	2.34 (0.86-6.36)	10.23 (4.85-21.59)^§^	1.87 (0.64-5.43)	4.56 (1.14-18.22)	2.77 (0.69-11.06)
***Life threatening composite***	11.55 (5.53-24-10)^§^	3.30 (0.85-12.78)	8.20 (4.39-15.30)^§^	1.55 (0.35-6.97)	5.30 (1.78-15-78)	3.31 (1.59-6.90)^§^
Pulmonary disorders	5.31 (4.09-6.91)^§^	2.07 (1.17-3.66)^§^	5.27 (4.09-6.79)^§^	2.12 (1.17-3.84)^§^	1.08 (0.57-2.09)	0.81 (0.45-1.48)
Bacterial infections	4.37 (2.91-6.56)^§^	1.34 (0.57-3.14)	4.60 (3.08-6.88)^§^	1.43 (0.65-3.13)	0.48 (0.16-1.99)	0.42 (0.05-3.72)
pH < 7.00	3.32 (1.95-5.66)^§^	1.41 (0.54-3.69)	2.05 (1.29-3.26)	0.78 (0.31-2.00)	7.30 (4.07-13.10)^§^	7.02 (4.13-11.95)^§^
BD > 12 mmol/L	1.04 (0.73-1.48)	0.49 (0.24-0.97)	0.80 (0.57-1.12)	0.35 (0.16-0.77)	4.04 (2.91-5.60)^§^	3.28 (2.01-5.37)^§^
Apgar < 7	4.56 (3.03-6.87)^§^	2.06 (1.16-3.67)^§^	3.17 (2.22-4.55)^§^	1.01 (0.62-1.64)	5.43 (3.21-9.16)^§^	5.00 (2.60-9.61)^§^
Other**	4.48 (3.88-5.18)^§^	1.05 (0.81-1.36)	4.31 (3.75-4.94)^§^	1.99 (1.62-2.43)^§^	1.41 (1.04-1.93)	1.12 (0.79-1.57)
***Non-life threatening composite***	3.21 (2.83-3.65)^§^	0.94 (0.75-1.18)	2.92 (2.59-3.29)^§^	0.92 (0.73-1.17)	2.05 (1.65-2.54)^§^	1.78 (1.42-2.22)^§^
***Overall composite***	3.35 (2.96-3.79)^§^	0.95 (0.76-1.19)	3.01 (2.68-3.39)^§^	0.89 (0.70-1.12)	2.10 (1.70-2.60)^§^	1.92 (1.45-2.25)^§^

Footnotes: Risk ratios adjusted by maternal age, maternal body mass index, gestational age at delivery, pregnancy at risk, parity, fetal presentation, number of fetuses, presence of previous CD, neonatal birth weight, grade of urgency (e.g. maternal or fetal compromise requiring immediate delivery).

RR, risk ratios; SVD, spontaneous vaginal delivery; AVD, assisted vaginal delivery; CD, caesarean delivery; VD vaginal delivery; Adj, adjusted; PPH, post-partum hemorrhage; TED, thromboembolic disease; BD, base deficit.

*Any other condition requiring Intensive Care Unit admission.

**Any other condition requiring Neonatal Intensive Care Unit admission in term neonates (37–42 weeks).

^§^p < .001.

We assessed the outcomes by mode of delivery with bivariate and multivariate analyses in order to control for all possible confounders that can be both related to the need of an operative delivery and to the increased risk of adverse outcomes.

Considering either SVD or VD (SVD plus AVD) as the reference, CD was associated with a significantly higher risk of endometritis-infection (adjusted RRs 4.74 and 4.33 respectively) and selective neonatal complications such as pulmonary disorders (adjusted RRs 2.07 and 2.12, respectively). The risk of Apgar score less than 7 at five minutes was higher in CDs than SVDs (adjusted RR 2.06), and any other condition requiring NICU admission in neonates at term occurred more frequently in CDs than VDs (adjusted 1.99). In regard to the “protective effect”, CD was associated with a better composite maternal outcome for life threatening complications than VD. However the difference was not significant if the comparison considered only SVD.

When compared with SVD, AVD had a significantly higher risk of major and minor PPH (adjusted RRs 2.41 and 1.41, respectively), III-IV degree tears (adjusted RRs 2.26) and life threatening, non-life threatening and overall composite adverse maternal outcomes (adjusted RRs 2.24, 1.57 and 1.67, respectively).

As for the neonate, AVD was associated with a higher risk of mortality within 28 days (adjusted RRs 7.12), arterial cord pH less than 7.00 and base deficit greater than 12 mmol/l (adjusted RRs 7.02 and 3.28, respectively), Apgar score less than 7 at five minutes (adjusted RR 5.00), and life threatening, non-life threatening and overall composite neonatal morbidities (adjusted RRs 3.31, 1.78 and 1.92 respectively).

### Multivariate Analysis of Maternal and Neonatal Outcomes According to Outlier Status

Adjusted maternal and neonatal outcomes according to the outlier status for CD and AVD are described in Tables 3 and 4, respectively.

**Table 3:** **Adjusted outcomes (individual and composite) by caesarean delivery rates outlier status**

	**Caesarean delivery outlier**		
	**Within expected**	**Above expected**	**Below expected**
**Maternal complications**	***% (95% CI)***	***% (95% CI)***	***% (95% CI)***
Major PPH	0.20 (0.20-0.21)	0.59 (0.57-0.62)	0.37 (0.35-0.39)
Hysterectomy	0.03 (0.03-0.04)	0.09 (0.07-0.11)	0.04 (0.03-0.04)
Wound hematoma	0.32 (0.31-0.33)	0.32 (0.31-0.33)	0.33 (0.32-0.33)
TED	0.07 (0.05-0.08)	0.03 (0.03-0.03)	0.03 (0.03-0.04)
Uterine rupture	0.01 (0.01-0.01)	0.01 (0.01-0.01)	0.01 (0.01-0.01)
***Life threatening composite***	0.53 (0.51-0.55)	0.92 (0.89-0.94)	0.71 (0.68-0.73)
Minor PPH	1.85 (1.81-1.89)	2.71 (2.65-2.77)	2.51 (2.47-2.56)
III-IV degree tears	0.28 (0.27-0.28)	0.35 (0.34-0.36)	0.34 (0.33-0.35)
Wound Dehiscence	0.17 (0.17-0.18)	0.16 (0.15-0.17)	0.16 (0.16-0.17)
Endometritis-Infection	0.44 (0.43-0.46)	0.52 (0.50-0.54)	0.42 (0.41-0.44)
Bowel or bladder injury	0.02 (0.02-0.03)	0.03 (0.03-0.03)	0.03 (0.03-0.03)
Anaesthesiological	0.05 (0.04-0.05)	0.05 (0.04-0.05)	0.05 (0.04-0.05)
Other*	0.09 (0.08-0.10)	0.16 (0.14-0.17)	0.12 (0.11-0.13)
***Non-life threatening composite***	2.46 (2.42-2.51)	3.82 (3.75-3.89)	3.20 (3.15-3.25)
***Overall composite***	2.97 (2.92-3.02)	4.69 (4.61-4.78)	3.90 (3.85-3.96)
**Neonatal complications**	***% (95% CI)***	***% (95% CI)***	***% (95% CI)***
Mortality <7 days	0.03 (0.03-0.04)	0.16 (0.14-0.19)	0.11 (0.09-0.13)
Mortality <28 days	0.05 (0.05-0.06)	0.13 (0.10-0.16)	0.08 (0.06-0.10)
Neurologic symptoms	0.06 (0.05-0.07)	0.40 (0.34-0.46)	0.21 (0.18-0.24)
***Life threatening composite***	0.13 (0.10-0.16)	0.53 (0.44-0.62)	0.23 (0.20-0.26)
Pulmonary disorders	0.66 (0.61-0.70)	2.24 (2.08-2.39)	1.60 (1.50-1.70)
Bacterial infections	0.46 (0.42-0.49)	0.94 (0.84-1.04)	0.58 (0.54-0.61)
pH < 7.00	0.66 (0.62-0.71)	0.35 (0.33-0.37)	0.48 (0.47-0.50)
BD > 12 mmol/L	1.22 (1.19-1.26)	1.30 (1.27-1.34)	1.41 (1.38-1.44)
Apgar < 7	0.59 (0.57-0.61)	0.99 (0.89-1.09)	0.70 (0.66-0.73)
Other**	1.74 (1.57-1.91)	8.20 (7.75-8.66)	4.71 (4.46-4.95)
***Non-life threatening composite***	3.56 (3.33-3.79)	9.08 (8.62-9.54)	6.13 (5.88-6.37)
***Overall composite***	3.85 (3.67-4.02)	9.66 (9.31-10.01)	6.29 (6.10-6.48)

Footnotes. Outcomes were adjusted by maternal age, maternal body mass index, gestational age at delivery, pregnancy at risk, parity, fetal presentation, number of fetuses, presence of previous CD, neonatal birth weight, grade of urgency (e.g. maternal or fetal compromise requiring immediate delivery) and cluster variables: centers with NICU and obstetric volume per center (number of deliveries/year).

PPH, post-partum hemorrhage; TED, thromboembolic disease; BD, base deficit.

*Any other condition requiring admission to Intensive Care Unit.

**Any other condition requiring admission to Neonatal Intensive Care Unit in term neonates (37–42 weeks).

**Table 4:** **Adjusted outcomes (individual and composite) by assisted vaginal delivery rates outlier status**

	**Assisted vaginal delivery outlier**		
	**Within expected**	**Above expected**	**Below expected**
**Maternal complications**	***% (95% CI)***	***% (95% CI)***	***% (95% CI)***
Major PPH	0.43 (0.41-0.45)	0.49 (0.47-0.51)	0.20 (0.19-0.22)
Hysterectomy	0.06 (0.05-0.07)	0.04 (0.03-0.04)	0.04 (0.03-0.05)
Wound hematoma	0.31 (0.31-0.33)	0.34 (0.33-0.36)	0.32 (0.31-0.34)
TED	0.04 (0.03-0.04)	0.03 (0.02-0.03)	0.06 (0.05-0.07)
Uterine rupture	0.01 (0.01-0.01)	0.02 (0.02-0.02)	0.01 (0.01-0.01)
***Life threatening composite***	0.76 (0.74-0.78)	0.80 (0.78-0.83)	0.53 (0.51-0.55)
Minor PPH	2.38 (2.34-2.42)	2.97 (2.91-3.04)	1.91 (1.86-1.95)
III-IV degree tears	0.32 (0.31-0.33)	0.39 (0.37-0.40)	0.29 (0.28-0.30)
Wound Dehiscence	0.17 (0.16-0.17)	0.15 (0.14-0.15)	0.17 (0.16-0.18)
Endometritis-Infection	0.46 (0.45-0.47)	0.46 (0.44-0.48)	0.43 (0.41-0.44)
Bowel or bladder injury	0.03 (0.03-0.03)	0.03 (0.02-0.03)	0.03 (0.02-0.03)
Anaesthesiological	0.04 (0.04-0.04)	0.05 (0.05-0.06)	0.05 (0.05-0.05)
Other*	0.13 (0.12-0.14)	0.14 (0.12-0.16)	0.10 (0.09-0.12)
***Non-life threatening composite***	3.22 (3.17-3.26)	3.83 (3.76-3.91)	2.45 (2.41-2.50)
***Overall composite***	3.96 (3.91-4.01)	4.61 (4.52-4.70)	2.97 (2.91-3.03)
**Neonatal complications**	***% (95% CI)***	***% (95% CI)***	***% (95% CI)***
Mortality <7 days	0.10 (0.08-0.11)	0.20 (0.17-0.23)	0.04 (0.03-0.03)
Mortality <28 days	0.07 (0.05-0.09)	0.16 (0.13-0.20)	0.06 (0.05-0.07)
Neurologic symptoms	0.19 (0.17-0.22)	0.47 (0.39-0.53)	0.05 (0.05-0.06)
***Life threatening composite***	0.22 (0.19-0.24)	0.65 (0.54-0.76)	0.10 (0.08-0.11)
Pulmonary disorders	1.46 (1.37-1.54)	2.65 (2.45-2.84)	0.66 (0.61-0.70)
Bacterial infections	0.55 (0.52-0.58)	1.10 (0.98-1.22)	0.43 (0.40-0.46)
pH < 7.00	0.48 (0.46-0.49)	0.36 (0.33-0.38)	0.65 (0.61-0.69)
BD > 12 mmol/L	1.31 (1.28-1.34)	1.50 (1.46-1.54)	1.26 (1.22-1.30)
Apgar < 7	0.70 (0.67-0.72)	1.03 (0.91-1.15)	0.61 (0.57-0.64)
Other**	5.04 (4.81-5.27)	7.83 (7.33-8.33)	1.65 (1.49-1.82)
***Non-life threatening composite***	6.34 (6.11-6.58)	9.02 (8.52-9.52)	3.37 (3.15-3.58)
***Overall composite***	6.52 (6.34-6.71)	9.77 (9.38-10.16)	3.52 (3.35-3.68)

Footnotes. Outcomes were adjusted by maternal age, maternal body mass index, gestational age at delivery, pregnancy at risk, parity, fetal presentation, number of fetuses, presence of previous CD, neonatal birth weight, grade of urgency (e.g. maternal or fetal compromise requiring immediate delivery) and cluster variables: centers with NICU and obstetric volume per center (number of deliveries/year).

PPH, post-partum hemorrhage; TED, thromboembolic disease; BD, base deficit.

*Any other condition requiring admission to Intensive Care Unit.

**Any other condition requiring admission to Neonatal Intensive Care Unit in term neonates (37–42 weeks).

With regard to caesarean deliveries, the “above” group had worse maternal outcomes if compared to the “within” reference group. The incidence of major and minor PPH, hysterectomy, III-IV degree tears, endometritis-infection, any other condition requiring admission to intensive care unit, as well as life threatening, non-life threatening and overall composite maternal adverse outcomes, was significantly higher in centers with above the expected CD rates. This group showed also significantly higher frequencies of almost all the neonatal complications (except for cord pH <7).

It is of interest to note that similar results were also observed in centers with CD rates below the expected (Table 3).

Higher rates of selected maternal complications (PPH, wound hematoma, uterine rupture, III-IV degree tears, anaesthesiological complications, and non-life threatening and overall composite maternal adverse outcomes) were also observed in centers with AVD rates above the expected. This group had also significantly higher rates of unfavorable neonatal outcomes for almost all the considered conditions.

Inversely, institutions with an AVD rate below the expected had significantly better maternal and neonatal outcomes than the “within” AVD rates institutions (Table 4).

## Discussion

There is a worldwide growing debate on quality assessment in obstetric care and this issue represents an important part of the National Health Systems (NHS) agenda [21-24].

Whether processes or outcome measures are used as markers of quality, an ideal assessment should encompass variables that are clinically relevant, easy to define and observe. Although the evaluation of CD rates – according to their adjusted rates – has been suggested as one of the most important indicators of quality, it has been criticized because of its controversial ability to capture both maternal and neonatal outcomes [8].

Our multicenter study is the first to determine the adjusted incidence of adverse maternal and neonatal outcomes according to institutional outlier status for both adjusted AVD and CD rates.

**Figure 2:**
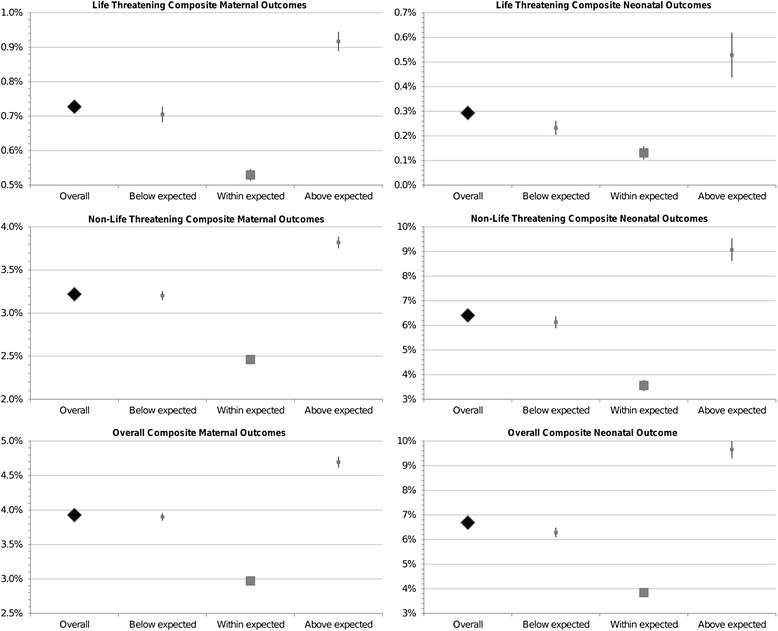
**Forest plots of life threatening, non-life threatening and overall composite maternal and neonatal complications, by caesarean delivery rates outlier status.**

**Figure 3:**
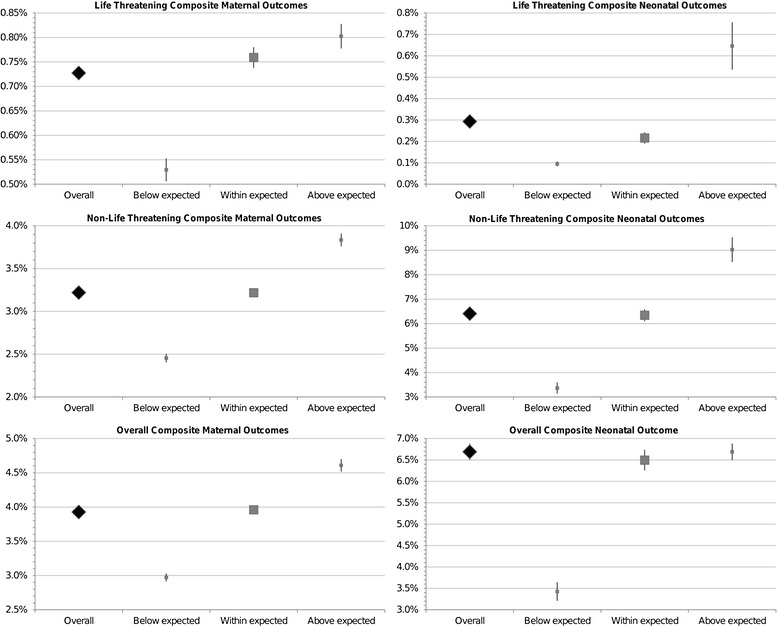
**Forest plots of life threatening, non-life threatening and overall composite maternal and neonatal complications, by assisted vaginal delivery rates outlier status.**

We observed that both centers with CD rates above or below the expected had a higher incidence of almost all the maternal and neonatal clinically significant adverse outcomes. Moreover, centers with higher-than-expected AVD rates showed higher incidence of complications, whereas those with a rate of AVD below the expected had a significantly lower rate of selected and composite maternal and neonatal outcomes (Figures 2 and 3).

These results are of clinical relevance. As first, both CD rates and AVD rates must be considered for a correct evaluation of the performance of every maternity unit. If assisted vaginal deliveries are not considered as part of the quality care assessment, the evaluation can be misleading. Centers with CD rates within the expected can in fact be thought to provide a good care, while they may actually dispense less optimal levels of care if their AVD rates are found to be higher-than-expected. The status of center G represents an example: the adjusted CD rate was within the expected and thus associated with “good outcomes”, but its “above” AVD rate was associated with an increased risk of complications. Second, both CD rates above and below the expected can be considered as an indicator of increased risk of maternal or neonatal morbidities. In this regard, it is clear that the best maternal and neonatal outcomes are offered by those institutions, as center E, that maintain a CD rate within the expected range and have a simultaneous low rate of AVD.

From our data, it seems that mode of delivery by itself cannot completely explain the differences in the most severe adverse maternal and neonatal outcomes as observed in different outlier status of operative deliveries.

In fact, if compared with SVD, as demonstrated in other studies [25], AVDs were associated with an increased risk of selected maternal and neonatal composite adverse outcomes. Caesarean deliveries, instead, increased only the risk of endometritis, newborn pulmonary disorders and Apgar less than 7 at five minutes.

The causal link between above and below the expected risk-adjusted CD rates and poorer maternal and especially neonatal outcomes is unclear. This relationship does not imply causality, but suggests that an association is present.

Despite the differences in study design, our results support the conclusions of Gould, Bailit, Srinivas: institutional CD rates both “above” or “below” the expected may be considered as indicators of increased risk of maternal or neonatal morbidities. Gould *et al*. focused their analysis only on outcomes of low risk pregnancies [5]. Bailit *et al.* evaluated the risk of adverse maternal and neonatal outcomes by considering only the outlier status for primary and not overall adjusted CD rates [6,7]. Srinivas *et al.* considered only selected measures of complications, such as maternal wound infection, post-partum hemorrhage, blood transfusion and neonatal mortality, asphyxia or seizures [8]. The main limitation of these studies was the model of risk adjusting outcomes. All of them based their analyses on retrospective collection of pregnancy data derived from birth certificates and hospital discharge records containing ICD-9 diagnoses codes. Moreover they did not consider relevant variables for risk adjustment such as, for example, maternal BMI, obstetric volume and conditions of impeding maternal or neonatal compromise. Medical records, birth certificates, diagnosis related group codes (DRG) and International Classification of Diseases - 9^th^ Revision (ICD-9) codes are commonly used as resources for research and quality surveillance in obstetric practice. However, these large datasets, which are usually used for other purposes such as for insurances or health statistics, often lack the information needed to homogenously risk-adjust the outcomes of interest for patient characteristics. [21,22,26,27]. Even though our study was not based on a large number of deliveries, it should be considered as one of the few in which the operative delivery rates and the incidence of maternal and neonatal complications were adjusted for unambiguous data. Information on maternal characteristics, antenatal obstetric conditions/risk factors and maternal/neonatal outcome variables was prospectively gathered in a dedicated database that allowed us to collect standardized and homogeneous data, excluding only 3.6% of the records from the final analysis because of missing data. Nevertheless our study, by prospectively collecting information on twelve maternal and ten neonatal adverse outcome variables, provided the information that overall CD rates – not only primary – may be considered as a measure of quality of care.

In regard to the association of outlier status for CD rates and neonatal morbidity, it might be hypothesized that increased morbidity observed in the “below” CD rate group might suggest that certain infants delivered vaginally could potentially have benefited from caesarean delivery. Alternatively, in these centers, an inappropriate delayed timing in the conduction of the delivery might have resulted in a higher rate of neonatal morbidity.

The increased rate of neonatal complications observed in the “above” CD rate group might be explained considering that the selection process in this group, though leading to more caesarean deliveries, failed to consider many cases that might have benefited from the caesarean delivery [5].

Moreover, strategies for managing labor and organizational models may vary between institutions and these might account for both different incidences of adverse outcomes and operative delivery rates [23,24,28].

Walsh *et al.* observed that both AVDs and CDs in the second stage of labor are associated with a similar increased risk of serious neonatal complications [29]. In our context, we may suppose that any inappropriate anticipation of an obstetric intervention in the second stage of labor, without respecting its “physiological” duration or without managing second stage according to the recommended guidelines, might increase the rate of both caesarean and operative deliveries and worsen the obstetric outcomes [22,24,28].

The literature does not clarify whether the hospital delivery volume might influence both the rate of operative deliveries and of maternal and neonatal complications [18-20,30]. In this regard, it is possible that smaller units might have a lower threshold for operative deliveries due to organizational reasons and lack of resources required to respond to medical emergencies. For the same reasons, these institutions could also present worse outcomes. This might not be the case of our study, because inter institutional variations in operative delivery rates and frequencies of adverse outcomes remained either between centers with less than 1000 deliveries/year and institutions with more than 1000 deliveries/year, despite the inclusion of obstetric volume, of type of neonatal organization (NICU availability) and delivery grade of urgency (emergency – no emergency) into the adjusted model. As suggested by Janakiraman *et al.*, it might be that the increased risk of maternal and neonatal complications could be related to hospital performance, independently from delivery volumes [20].

Despite the clinically relevant conclusions, we are aware that our study has its limitations. First, we did not consider separately every antenatal risk factor, labeling the pregnancy as “at risk” according to selected groups of risk conditions. However, other studies adopted this classification considering that a successful model for adjusting assisted delivery rates should consider the most relevant risk factors that must be acceptable to practicing obstetricians [21-23,31]. Second, we did not include other variables, such as race/ethnicity or socio-economic status or habits (e.g. smoking), in the risk adjustment. However, the former was not assessed because of the very low prevalence of non-Caucasians in our region and considering this variable should not have a relevant role in the prediction of operative delivery [32]; the latter was not considered because the collected data included all the clinical adverse conditions that are associated with “bad” habits (e.g. intrauterine growth restriction, preterm delivery). Third, there is no wide agreement on which indicators of outcome need to be evaluated to assess obstetric quality. In this regard, we considered the short term clinically meaningful indicators that are included in the Agency for Health Care Research and Quality report, in the Adverse Outcome Index and in the recent model proposed by Sibanda *et al.* on behalf of the Royal College of Obstetricians and Gynaecologists [33-36]. Finally, a further limitation of our study was the inability to assess what factors contributed to adverse outcomes in the outlier settings. In this context caesarean and assisted vaginal deliveries might reflect the differences to a selected processes of care (e.g. training, adherence to guidelines) that might explain inter-institutional variation of outcomes [30]. Nonetheless the aim of our study was not to measure the process of care, but to evaluate whether variations of both CD and AVD rates among institutions could explain differences in outcomes.

## Conclusions

Our results support the belief that evaluating the CD rates without taking into account the AVD rates might not provide a reliable view of obstetric performance. In this context, the case-mix adjustment for a complete and standardized set of variables and the knowledge of the outlier status for both assisted vaginal and caesarean deliveries are crucial to properly assess the level of care among institutions, giving the opportunity to modify the management and improve the outcomes [4,37].

However we are aware that more research is required to develop a consensus about accepted, reproducible and clinically relevant indicators of maternal and neonatal outcomes that need to be evaluated in the process of labor audit [38].

## Abbreviations

Adj: Adjusted
AVD: Assisted vaginal delivery
BD: Base deficit
BMI: Body mass index
CD: Caesarean delivery
ICU: Intensive care unit
NICU: Neonatal intensive care unit
PPH: Post-partum hemorrhage
RR: Risk ratio
SVD: Spontaneous vaginal delivery
TED: Thromboembolic disease
VD: Vaginal delivery
